# Enhancement of bZIP60 function through C-terminal region translated after splicing in *Arabidopsis*

**DOI:** 10.5511/plantbiotechnology.25.0603a

**Published:** 2025-12-25

**Authors:** Yuji Iwata, Hiroyuki Mizoguchi, Nozomu Koizumi

**Affiliations:** 1 Graduate School of Agriculture, Osaka Metropolitan University, 1-1 Gakuen-cho, Naka-ku, Sakai, Osaka 599-8531, Japan

**Keywords:** *Arabidopsis thaliana*, bZIP transcription factor, endoplasmic reticulum, nuclear localization signal, unfolded protein response

## Abstract

The unfolded protein response (UPR) is a central regulatory pathway that ensures the proper function of the endoplasmic reticulum (ER) through efficient protein folding and quality control. In Arabidopsis, *bZIP60* mRNA is activated by an IRE1-mediated unconventional splicing that excises a 23-nucleotide intron, resulting in the spliced form (*bZIP60s* mRNA) that encodes the active bZIP60 transcription factor lacking a transmembrane domain. In this study, we investigated the functional role of the spliced form-specific C-terminal extension, hereafter referred to as ORF2. Transient expression assays in Arabidopsis mesophyll protoplasts demonstrated that full-length bZIP60s potently activates the *BiP3* promoter compared to a truncated variant lacking ORF2. Fusion of ORF2 to transcription factors unrelated to the UPR did not enhance their transcriptional potency, underscoring its specialized role in the context of bZIP60s. Furthermore, mutation in a conserved nuclear localization signal within ORF2 decreased promoter activation by bZIP60s. Fusion of ORF2 to GFP enhanced the nuclear localization of GFP. Our results suggest that ORF2 is critical for the full transcriptional activity of bZIP60s to ensure an efficient UPR.

## Introduction

The unfolded protein response (UPR) is a highly conserved signaling pathway that helps eukaryotic cells maintain endoplasmic reticulum (ER) homeostasis. In the face of adverse conditions, such as high secretory demand, heat shock, or pathogen infection, misfolded or unfolded proteins can accumulate in the ER. To mitigate this protein-folding stress, the UPR coordinates transcriptional changes that restore ER function, thereby promoting cell survival and adaptation (Walter and Ron 2011).

In mammals, three membrane- tethered ER stress sensors percept ER stress: IRE1, PERK and ATF6. Of these, IRE1, highly conserved in various organisms, detects the folding status of proteins by the sensor domain in the ER lumen and activates its RNase domain in cytoplasmic side via autophosphorylation under the ER stress condition. The activated RNase cleaved mRNA encoding bZIP transcription factors that enhance the transcription of the UPR related genes. Cleaved mRNA molecules are joined by tRNA ligase resulting generation of active bZIPs. This splicing is referred to as cytoplasmic splicing or unconventional splicing since the mechanism is different from ordinary splicing observed in the nucleus.

After cytoplasmic splicing, bZIPs are activated due to alternation of amino acid sequence. However, the mechanism of activation is quite different among organisms. In yeast, the bZIP, HAC1 gains nine amino acids due to change of stop codon position (Sidrauski and Walter 1997). Human XBP1 becomes a larger protein because of frameshift. XBP then translocates to the nucleus and functions as a transcription factor (Yoshida et al. 2001). Arabidosis bZIP60 is the target of IRE1 and activated, as described below.

**Figure 1:**
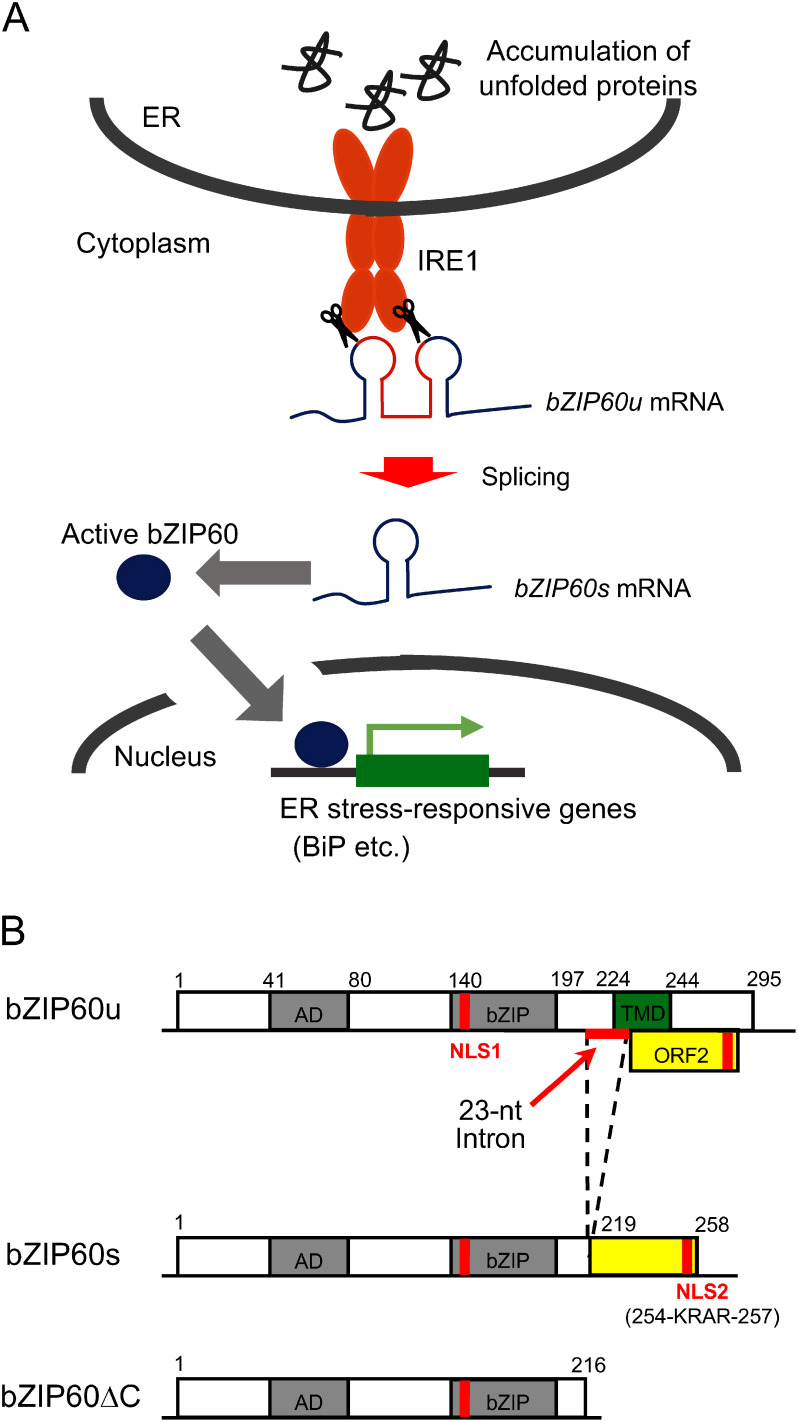
Figure 1. Schematic representation of IRE1-bZIP60 pathway in Arabidopsis. (A) Cytoplasmic splicing of *bZIP60* mRNA by IRE1. IRE1 recognizes and cleaves two stem-loops of *bZIP60u* mRNA, resulting in *bZIP60s* mRNA that encodes the nuclear localized, active bZIP60s protein. (B) Unspliced and spliced forms of *bZIP60* mRNAs and their encoded protein products. bZIP60ΔC used in this study was also shown. NLS1 in the bZIP domain and NLS2 in ORF2 are indicated by vertical red bars. AD, activation domain; TMD, transmembrane domain.

bZIP60 discovered in Arabidopsis (Iwata and Koizumi 2005) is a central transcription factor in the plant UPR that is conserved in a wide range of plant species (Deng et al. 2011; Hayashi et al. 2012; Li et al. 2020; Nagashima et al. 2011; Takeda et al. 2022). It is initially produced as an unspliced mRNA (*bZIP60u* mRNA) encoding a membrane-tethered protein (Iwata et al. 2008). Upon ER stress, *bZIP60u* mRNA is selectively cleaved by IRE1 (Figure 1A). It removes a 23-nucleotide intron from *bZIP60u* mRNA in Arabidopsis, resulting in a spliced mRNA (*bZIP60s* mRNA) and causing a translational frameshift that yields bZIP60s protein (Deng et al. 2011; Nagashima et al. 2011, 2016). Unlike the unspliced form, bZIP60s lacks the transmembrane domain and instead contains a novel C-terminal region (Figure 1B), allowing it to translocate to the nucleus and activate target genes crucial for restoring ER homeostasis, including *BiP* and *CNX* (Iwata and Koizumi 2005; Iwata et al. 2008), which encode molecular chaperones that assist in protein folding and quality control (Deng et al. 2011; Hayashi et al. 2012; Li et al. 2020; Nagashima et al. 2011; Takeda et al. 2022).

It has been demonstrated that bZIP60u and bZIP60s are localized to the ER and the nucleus, respectively (Deng et al. 2011; Iwata et al. 2008). It is clear that removal of the transmembrane domain is critical for the nuclear entry of bZIP60s because the truncated bZIP60u protein consisting of 1–216 aa (hereafter referred to as bZIP60ΔC), which lacks the C-terminal region containing the transmembrane domain, was localized to the nucleus (Iwata and Koizumi 2005), probably owing to the nuclear localization signal (NLS) in the basic leucine-zipper (bZIP) domain (Figure 1B). It has also been shown that bZIP60ΔC activated *BiP* and *CNX* promoters in a transient assay (Iwata and Koizumi 2005). Indeed, bZIP60ΔC contains both the DNA-binding bZIP domain and the transactivation domain (Iwata et al. 2009) (Figure 1B). Nevertheless, the newly encoded C-terminal segment, hereafter referred to as ORF2 (Figure 1B), has been suggested to have important functional properties. ORF2 contains two NLSs, and the data suggests that one of them could act as a functional NLS (Zhang et al. 2015).

In this study, we used transient expression assays in Arabidopsis mesophyll protoplasts to elucidate the roles of bZIP60 ORF2. By examining the transcriptional activation of the *BiP3* promoter, we dissected how mutations in ORF2, particularly in the conserved serine residue and the NLS motif, affect bZIP60s activity and localization. We also tested whether fusing ORF2 to other transcription factors could enhance their transcriptional potency, thereby determining if ORF2 functions as a general activation module or has a more specialized role in bZIP60s.

## Materials and methods

### Plant materials

We used *Arabidopsis thaliana bzip60-1* mutant in Col-0 background previously reported (Iwata et al. 2008). Arabidopsis plants were grown on half-strength MS medium (Murashige and Skoog 1962) at 22°C in 16-h light/8-h dark cycle for four weeks.

### Plasmid construction

Vectors expressing Arabidopsis wild-type and mutant variants of bZIP60 were generated as follows. bZIP60u, bZIP60s, and bZIP60ΔC, all with 3×FLAG-coding sequence at their 5′ ends, were PCR amplified, and introduced into the downstream of the CaMV 35S promoter in the modified pBI221 vector using XhoI and SpeI sites. Mutation was introduced by PCR based site-directed mutagenesis to produce the S246A mutant variant of bZIP60. The ORF2ΔNLS2 variant, in which the KRAR sequence of 254–257 aa in bZIP60s was substituted for four consecutive alanine residues, was generated by gene synthesis. For MYB58-ORF2-expressing constructs, MYB58 and ORF2 cDNA fragments were amplified using MYB58 and bZIP60s cDNA clones as templates, and two fragments were subsequently joined by overlapping PCR, which was then inserted downstream of CaMV 35S promoter between BsrGI and SacI sites. NST3-ORF2-expressing constructs were similarly prepared, except that KpnI site was used instead of SacI site. OFR2 (219–258 aa of bZIP60s) and ORF2ΔNLS were PCR amplified and cloned into the 3′ end of the GFP-coding sequence of pBI221-GFP vector using BsrGI and SacI sites. Nucleotide sequences of all the plasmids generated in this study were confirmed by Sanger sequencing. All the primers used to generate these plasmids are listed in Supplementary Table S1.

BiP3pro-Luc and CNX1pro-Luc reporter and 35Spro-RLuc reference constructs were previously described in Iwata and Koizumi (2005). Approximate 1,000 bp promoter regions of *CCoAOMT1* and *MYB46* were amplified and cloned into pDONR_P4P1R and then R4L1pDEST_GLHSP vector (Fujiwara et al. 2014) by the GATEWAY reactions (Themo Fisher Scientific Inc.). *MYB58* and *NST3* were amplified and cloned into pDONR207 and then pDEST35SHSP vector (Sakamoto et al. 2022) by the GATEWAY reactions.

### Transient assay

Leaf mesophyll protoplasts were prepared from Arabidopsis *bzip60-1* mutant plants as described in previous studies (Wu et al. 2009; Yoo et al. 2007). Protoplasts were transfected with 3 µg of a BiP3pro-Luc or CNX1pro-Luc reporter plasmid, a 35Spro-bZIP60, 35Spro-MYB58, or 35Spro-NST3 effector plasmid, and a 35Spro-RLuc reference plasmid, and incubated for 22°C for 16 h.

Luciferase activity measurements were carried out using Dual-Luciferase Reporter Assay System (Promega). Protoplasts were suspended with 1× Passive Lysis Buffer, and the crude protein extracts were subjected to dual-luciferase assay. Firefly luciferase activity was normalized to Renilla luciferase activity.

For immunoblot analysis, protein extracts used for dual-luciferase assays were fractionated by SDS-PAGE with 10% polyacrylamide gel and transferred to PVDF membrane (Millipore). Anti-DDDDK-tag mAb-HRP-DirecT (MBL) with a dilution factor of 1 : 10,000 and Immobilon Forte Western HRP (Millipore) were used as a primary antibody and a substrate, respectively, to detect 3×FLAG-bZIP60 variants. Signal intensities were quantified using the ImageJ software.

Image acquisition of GFP fluorescence of protoplasts expressing GFP was performed using an LSM700 confocal laser scanning microscope (ZEISS).

## Results

### bZIP60s activated the *BiP3* promoter more strongly than bZIP60ΔC

**Figure 2:**
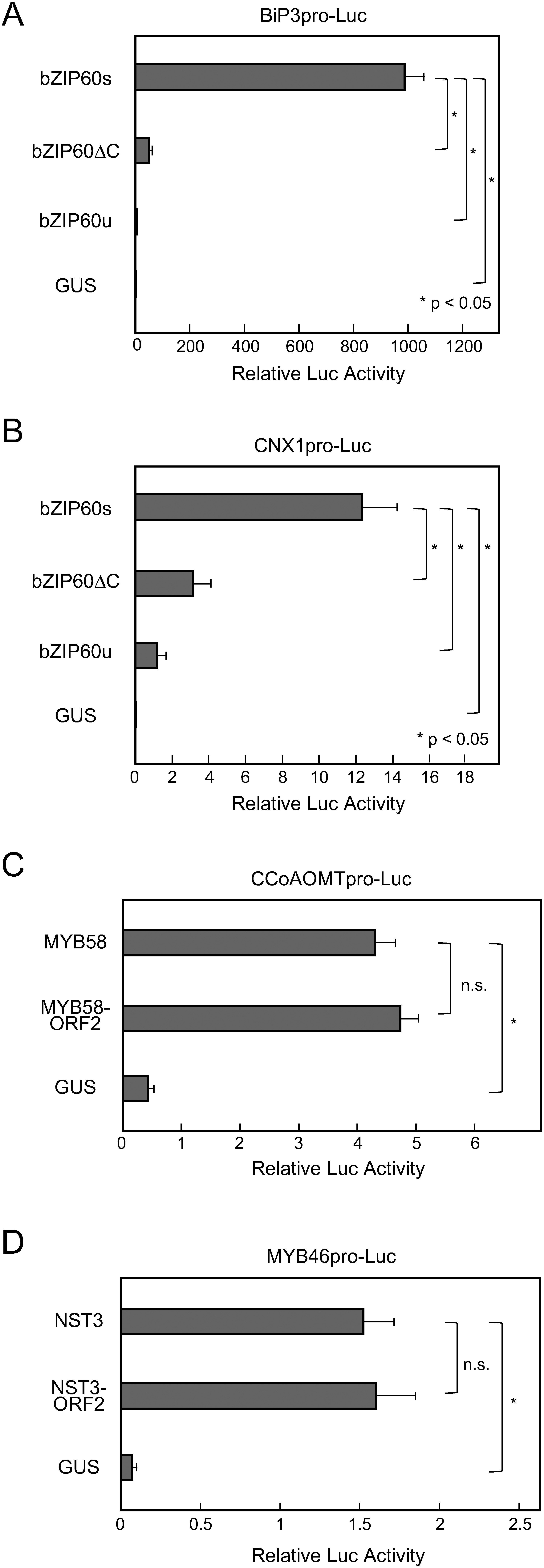
Figure 2. Effect of bZIP60 ORF2 on promoter activation in a transient assay. (A, B) bZIP60u, bZIP60s, or bZIP60ΔC was co-expressed with BiP3pro-Luc (A) or CNX1pro-Luc (B) constructs and dual-luciferase assays were carried out. GUS was expressed as a negative control effector. (C) MYB58 or MYB58-ORF2 was co-expressed with CCoAOMTpro-Luc construct, and dual-luciferase assays were carried out. (D) NST3 or NST3-ORF2 was co-expressed with MYB46pro-Luc construct, and dual-luciferase assays were carried out. Luciferase activities were normalized by Renilla luciferase activities. The data are means with SE from three independent experiments. Asterisks represent a significant difference by Welch’s *t*-test.

To examine whether the C-terminal region unique to bZIP60s (i.e., ORF2) contributes to transcriptional activation, we compared the activity of full-length bZIP60s with a truncated version lacking the ORF2 region (bZIP60ΔC). The bZIP60 variants were tagged with a FLAG epitope at their N-terminus to enable detection at the protein level. In Arabidopsis mesophyll protoplasts, bZIP60s strongly induced *BiP3* and *CNX1* promoter activity, whereas bZIP60ΔC showed significantly reduced activity (Figure 2A, B). These results indicate that the ORF2-encoded portion of bZIP60s is required for robust transcriptional activation of *BiP3*.

### ORF2 did not enhance transcriptional activation when fused to other factors

To test whether ORF2 serves as a generic transactivation domain, we examined whether ORF2 has a positive effect on the transactivation activity of transcription factors unrelated to the UPR. We chose two Arabidopsis transcription factors, MYB58 (Zhou et al. 2009) and NST3 (McCarthy et al. 2009; Mitsuda et al. 2007). We fused the ORF2 region to the C-terminus of MYB58 and NST3 and examined whether ORF2 enhances the transcriptional activation of MYB58 and NST3 in a protoplast assay. Unlike the dramatic enhancement observed with bZIP60s, the addition of ORF2 did not increase the transcriptional activity of MYB58 or NST3 on their target promoters (*CCoAOMT* promoter for MYB58, *MYB46* promoter for NST3) (Figure 2C, D). This result suggests that ORF2 does not act as an autonomous activation domain capable of broadly enhancing transcription but may have a more specialized role in the context of bZIP60s.

### ORF2 requires NLS but not Serine 246 for its function on transcriptional activation

**Figure 3:**
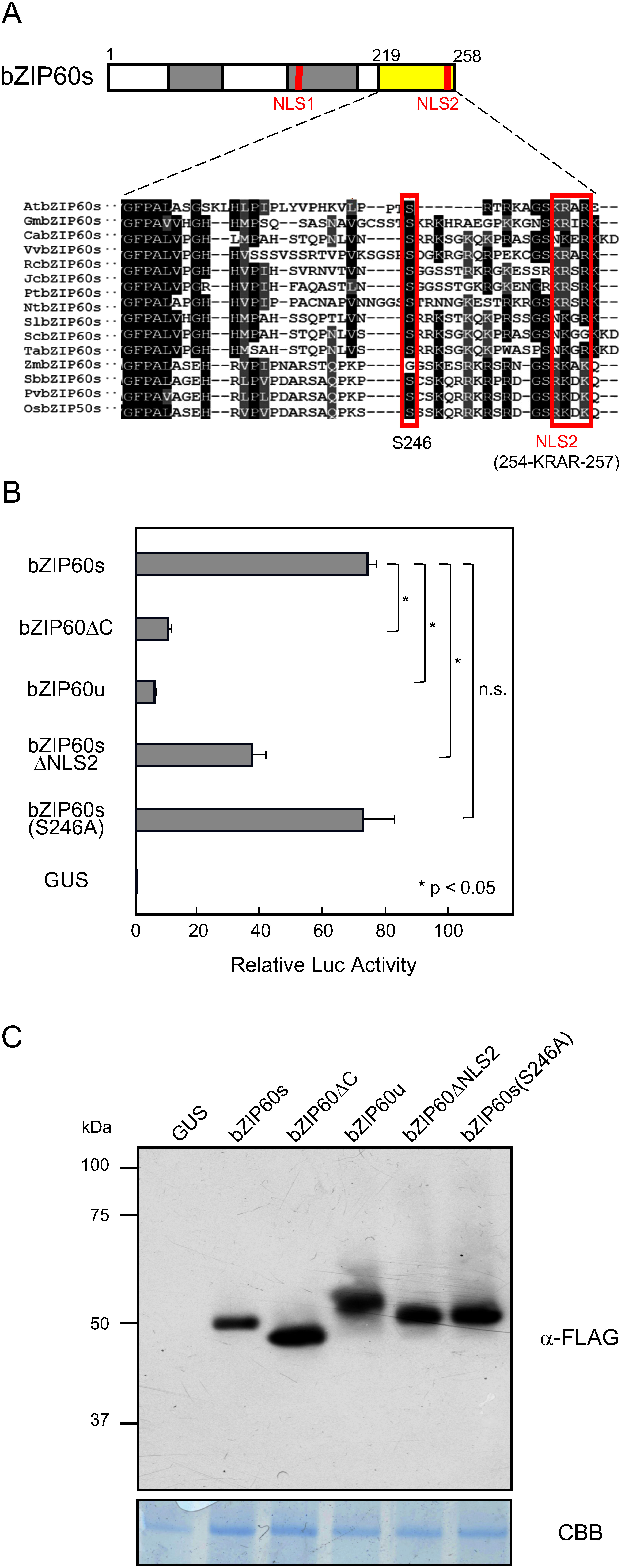
Figure 3. Effect of mutation in bZIP60 ORF2 on promoter activation. (A) Amino acid sequence alignment of bZIP60s ORF2 in different plant species. A conserved NLS and a serine residue at position 246 of Arabidopsis are indicated. (B) bZIP60s protein variants with mutations in ORF2 were co-expressed with BiP3pro-Luc constructs, and dual-luciferase assays were carried out as in Figure 2A. Luciferase activities were normalized by Renilla luciferase activities. The data are means with SE from three independent experiments. Asterisks represent a significant difference by Welch’s *t*-test. (C) Proteins were extracted from Arabidopsis protoplasts that express various bZIP60 variants and subjected to immunoblot analysis using anti-FLAG antibodies. A Coomassie blue-stained gel was presented as a loading control.

NLS is present at the C-terminal end of ORF2 in Arabidopsis bZIP60 (Figure 1B), and this is also observed in ORF2 of other plant bZIP60s proteins (Figure 3A). An amino acid sequence alignment also revealed that the serine residue at position 246 of ORF2 of Arabidopsis bZIP60 is conserved among dicots and monocots, raising the possibility that this site is important for the function of ORF2. Therefore, we generated two bZIP60s variants; bZIP60sΔNLS2, in which the KRAR sequence of NLS2 is substituted for four consecutive alanine residues, and bZIP60s(S246A), a serine-to-alanine mutant at position 246. We tested their effects on bZIP60s activation of the *BiP3* promoter. bZIP60sΔNLS2 exhibited roughly half the *BiP3* promoter activation of wild-type bZIP60s, whereas bZIP60s(S246A) activated the *BiP3* promoter at levels comparable to wild-type bZIP60s (Figure 3B). This indicates that ORF2-encoded NLS2 contributes to full transcriptional activation of the UPR targets, while the serine residue at position 246 is not essential under our assay conditions.

### Mutation in Ser246 or the NLS do not affect protein levels of bZIP60s

To examine whether the differences in promoter activity are due to differential protein abundance, we analyzed the protein accumulation of wild-type and mutant bZIP60s, along with bZIP60u and bZIP60ΔC, in protoplasts. Immunoblot analysis revealed that the differences in protein levels were relatively small compared to those observed in the luciferase assay (Figure 3C), indicating that the reduced activation by the NLS mutant and the unaltered activity by the S246A mutant are not attributable to changes in protein stability or accumulation.

### ORF2 increases nuclear localization in GFP fusion constructs

**Figure 4:**
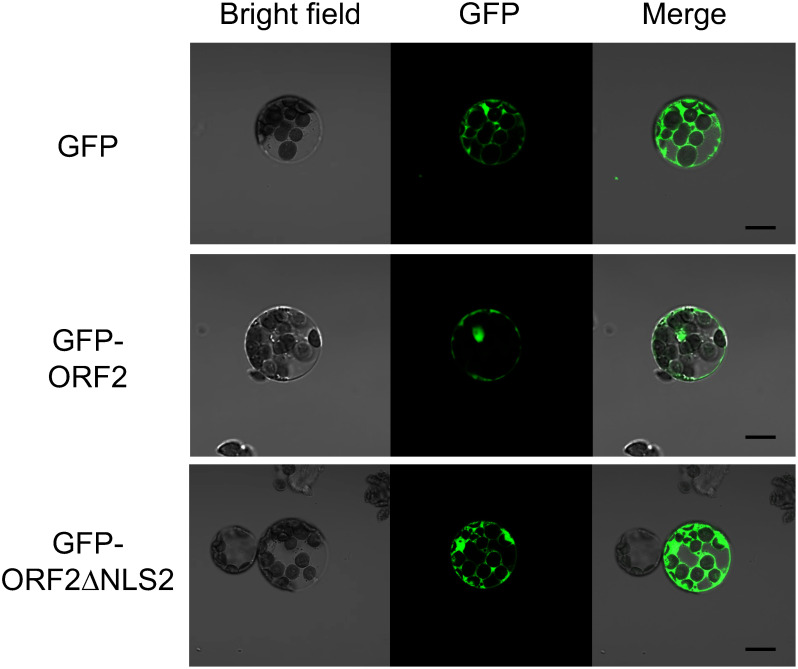
Figure 4. Effect of bZIP60 ORF2 on nuclear localization. Arabidopsis protoplasts expressing GFP, GFP fused with ORF2 (GFP-ORF2), and GFP fused with ORF2 variant with mutated NLS2 (GFP-ORF2ΔNLS2) were examined under the confocal laser scanning microscope. Bars=10 µm.

To examine ORF2’s role in subcellular localization, we fused ORF2 to the C-terminus of GFP. Compared to GFP alone, protoplasts expressing GFP-ORF2 exhibited stronger nuclear fluorescence and weaker cytosolic fluorescence (Figure 4). On the other hand, GFP fused to the NLS-dead ORF2 mutant did not display pronounced nuclear enrichment (Figure 4). These findings indicate that ORF2 has an ability to promote nuclear localization.

## Discussion

In this study, we provide evidence that the spliced form-specific ORF2 sequence in Arabidopsis bZIP60s is critical for full transcriptional activation of the UPR genes *BiP3* and *CNX1*. Although previous works established that bZIP60s is localized to the nucleus, the distinct contribution of ORF2 has remained understudied. Our results clarify that ORF2 is neither a simple transactivation domain when grafted onto transcription factors unrelated to the UPR, nor is its conserved serine residue critical for UPR target gene activation. Instead, the NLS at the C-terminus of ORF2 contributes to transcriptional activation.

The strong activation of the *BiP3* promoter by bZIP60s compared with a truncated bZIP60 lacking ORF2 indicates that ORF2 provides more than just the removal of the TMD and that its presence is essential for maximal promoter induction. Moreover, the inability of ORF2 to boost the activation potential of MYB58 or NST3 suggests that the domain’s regulatory role is specifically tailored to bZIP60s. One possible interpretation is that bZIP60s requires intramolecular interactions or specialized cofactor binding that depends on the bZIP domain plus ORF2, rather than ORF2 alone functioning as a universal activation domain.

Our findings also highlight that Ser246 is dispensable for bZIP60s activation under transient assay conditions, despite its high conservation. This result does not exclude the possibility that Ser246 might become phosphorylated in vivo under certain stress conditions or developmental stages, or that it may facilitate interactions with other components of the UPR machinery. Future work using phospho-specific antibodies or more detailed time-course experiments in stressed seedlings could uncover a conditional role for Ser246 in bZIP60s regulation.

In contrast, the NLS within ORF2 plays a clear functional role. Mutation of this NLS in bZIP60s resulted in a reduction in *BiP3* promoter activation and diminished nuclear accumulation of GFP-ORF2. Because the bZIP60ΔC is localized to the nucleus (Iwata and Koizumi 2005), the ORF2-encoded NLS likely augments this import pathway, ensuring that the majority of bZIP60s enters the nucleus to activate UPR target genes. Under physiological ER stress conditions, this enhanced nuclear import might be particularly critical for mounting a rapid and robust response. Besides nuclear localization, ORF2 might also be important for interactions with other proteins since bZIP60 has been reported to interact with other proteins (Kim et al. 2022; Pastor-Cantizano et al. 2020; Song et al. 2015).

In summary, our work uncovers an important role for the spliced form-specific ORF2 domain in Arabidopsis bZIP60s in transcriptional activation of UPR genes. These insights advance our understanding of the plant UPR, revealing that splicing of bZIP60 not only removes the ER membrane-targeting domain but also confers a specialized C-terminal extension for robust function.

## Abbreviations

bZIP: basic leucine-zipper
ER: endoplasmic reticulum
NLS: nuclear localization signal
UPR: unfolded protein response

## Description of supplementary files

Supplementary Table S1. Nucleotide sequences of primers used in this study.
