# Supplementary material for: Enhancement of bZIP60 function through C-terminal region translated after splicing in Arabidopsis
Source: Plant Biotechnol (Tokyo). 2025 Dec 25;42(4):383–8. doi: 10.5511/plantbiotechnology.25.0603a (PMC12781897; doi:10.5511/plantbiotechnology.25.0603a)
Supplement: Supplementary Data [file plantbiotechnology-42-4-25.0603a-s001.pdf]

Supplementary Table S1. Nucleotide sequences of primers used in this study.

Name	Nucleotide sequence (5' – 3')
ATG-3xFLAG-F-XhoI	AGCTCGAGATGGACTACAAAGACCATGACG
bZIP60U-R888-SpeI	AGACTAGTTCACGCCGCAAGGGTTAAGATTTG
bZIP60s-R777-SpeI-2	AGACTAGTTCACTCCCCGAGCCCGTTTAGAAC
bZIP60-R648-stopSpeI	AGACTAGTTCAAGACTCCTGCTTCGACATCATGG
NST3-F-BamHI	GGGGGGATCCATGGCTGATAATAAGGTC
NST3-nORF-R	GCAGGGAACCCTACAGATAAATG
NST3-nORF-F	CATTTATCTGTAGGGTTCCCTGCTTTGGCTTCTGGG
nORF-R-KpnI	AAAGGGTCCCTACTCCCGA
MYB58-F-BamHI	AAAGGGATCCATGGGCAAAGGAAG
MYB58-nORF-R	GCAGGGAACCCATGTATGAGGAGCTCGTA
MYB58-nORF-F	CCTCATACATGGGTTCCCTGCTTTGGC
nORF-R-SalI	AAAGGTCGACCTACTCCCGAGCCCGTTTA
BsrGI-ORF2-F	ACATGTACAAGGGGTTCCCTGCTTTG
SacI-ORF2dNLS-R	GCAGCTGAGTAGGAGCTCGAAT
